# Risk Perception and Communication, Plugged‐In: Fifty Years of Process

**DOI:** 10.1111/risa.70205

**Published:** 2026-02-19

**Authors:** George W. Warren, Sarah M. Duckett

**Affiliations:** ^1^ European Centre for Environment and Human Health University of Exeter Penryn UK; ^2^ Department of Geography King's College London London UK

**Keywords:** risk, risk communication, risk management, risk perception

## Abstract

Thirty years after Baruch Fischhoff's (1995) influential article *Risk Perception and Communication Unplugged*, this commentary revisits the developmental stages and conclusions outlined in this seminal paper documenting and reflecting on the development of risk communication. Fischhoff's work has shaped decades of research and practice, and while not intended in this way, many institutional efforts continue to rely on the earliest, technically focused stages. We reflect on how the field has evolved in the context of Fischhoff's stages, considering the impact of digital platforms, participatory methods, and societal change since 1995 that shape risk communication today.

Building on contemporary developments and our own experiences, we propose reordering Fischhoff's stages by moving partnership to the start of the communication process. We also revise its language to better reflect inclusive, collaborative, and dialogic approaches. Key themes include the persistence of information deficit models, the ethical responsibilities of communicators, and the tensions between theory and practice in risk communication. We also reflect on a selection of Fischhoff's (1995) conclusions, identifying still‐existing, and evolving issues arising 30 years later.

We argue that risk communication must be reconceptualized as a relational and political practice: One that respects public agency and centers partnership, rather than serving narrow persuasive aims. Fischhoff's contribution remains inspirational, provocative and instructive. It also challenges risk communicators to think more critically about the field's future and our responsibility to communicate risk ethically, inclusively, and meaningfully.

## 1 Introduction

In 1995, Baruch Fischhoff published the article titled *Risk Perception and Communication Unplugged: Twenty Years of Process* in *Risk Analysis* (Fischhoff 1995), detailing his personal reflections and experience of risk communication conducted over the previous 20 years. In the 30 years since, it has become a seminal reference within the risk communication community, cited almost two thousand times and inspiring research agendas, teaching materials, and risk communication strategies.

The most influential part of the article centers around the “Developmental Stages in Risk Management” (Fischhoff's stages) (Fischhoff 1995, 138), which describes the evolution of risk communication over the time period 1975–1995 into a series of “unsubstantiated empirically” (Breakwell 2007, 131), yet deeply personal and reflective stages (Table 1). While Fischhoff explicitly stated that these were developments in the field of risk communication, these stages have seemingly been reinterpreted as prescriptive stages in the risk communication process. Thirty years after its publication and subsequent use by risk communicators and researchers, much has changed.

**TABLE 1:** Table outlining Fischhoff's (1995) Developmental Stages in Risk Management.

All we have to do is get the numbers right
All we have to do is tell them the numbers
All we have to do is explain what we mean by the numbers
All we have to do is show them they've accepted similar risks in the past
All we have to do is show them that it's a good deal for them
All we have to do is treat them nice
All we have to do is make them partners
All of the above

This commentary draws on our reflections, aiming to understand and evaluate the validity and applicability of Fischhoff's (1995) developmental stages and conclusions, and their use in the field of risk communication, in the context of 30 years of developments in research, technology, and society. We evaluate Fischhoff's stages, followed by reflections on a selection of conclusions from the original article and how these relate to questions and debates on the current state of the discipline. This commentary serves as a celebration of the 30th anniversary of a seminal journal article published in *Risk Analysis*, and a reflection on the development and future of the risk communication discipline.

## 2 Fischhoff's (1995) Developmental Stages In Risk Management, 30 Years On

Fischhoff's (1995) stages present a somewhat tongue‐in‐cheek, but useful, history of and reflection on the practice of risk communication relating to public acceptance of risks (see Table 1). While models and conceptualizations of risk communication strategies have been developed before and since (Breakwell 2007; Leiss 1996; Renn 1992; Wardman 2008), these developmental stages have been interpreted and used prescriptively despite being descriptive in nature. Despite not being its intention, Fischhoff's stages present a clear, simple, and often‐followed guide for risk communication strategies (see Bouder 2010; Bourrier 2018; Wardman 2020). Importantly, Fischhoff did not number the stages in his table, but did in his discussion and interpretation, possibly inadvertently creating a numbered guide for risk communication stages and leading to a more prescriptive interpretation in non‐academic practice.

From our observations, and those noted by Spiegelhalter (2017), many risk communication practitioners still predominantly operate within the first three stages of Fischhoff's (1995) stages: *Getting the numbers right*, *telling people the numbers*, and, to a limited extent, *explaining what the numbers mean*. These stages are most familiar, institutionally supported, and technically bounded. Examples since 1995 include Swedish authorities’ Acrylamide risk communication strategy, and many nations’ and health authorities’ communications strategies during the COVID‐19 pandemic (Boholm 2019a; Warren and Lofstedt 2021). They continue to reflect top‐down, information‐deficit models of communication, where information is conceptualized as flowing unidirectionally from expert to public (Burgess et al. 1998; Lofstedt 2003; Owens 2000). This model assumes that improving clarity or accessibility of data alone will be enough to address concerns or build trust, despite an abundance of evidence to the contrary (Simis et al. 2016; Suldovsky 2017). The subsequent fourth and fifth stages, *showing them they've accepted similar risks in the past* and *showing them it's a good deal for them*, also reflect a lack of interest in public views in a presumed unidirectional transfer of information from those with power and expertise to those often shouldering much of the risk.

However, this narrow focus on “getting the message right” is increasingly inadequate (Bearth and Siegrist 2022; Renn 2014; Zhu and Lejano 2024). Risk communication practitioners today must contend with contested knowledge, shifting authority, and complex social dynamics. Compared to 1995, the rise of social media and decentralized forms of information sharing have increased individual and organizational ability to create, disseminate, and consume information (Krimsky 2007; Pascual‐Ferrá et al. 2022; Wendling et al. 2013). Solely focusing on technical accuracy or numerical literacy overlooks the relational and political dimensions of risk, where issues of trust, legitimacy, and inclusion play a decisive role.

As a result, the latter of Fischhoff's (1995) stages are often underdeveloped in risk communication approaches, particularly in practice despite much research on the topic over the last 30 years. While some examples of *treating them nice* and *making them partners* exist, these practices are frequently sidelined. This creates a lopsided view, where risk communicators all too often see “the public” as passive recipients of information rather than active participants in shaping how risks are defined, understood, and managed.

We therefore pay particular attention to these latter stages within our commentary. Responding to this power imbalance between communicator and publics, we propose that any use of Fischhoff's stages as a more prescriptive guide to risk communication must consider reorganizing the developmental stage relating to partnership earlier in the process (see Table 2). Not because publics have become easier to engage, but because partnership, public support, and trust have become more central to mitigating risks and become more complex, alongside other long‐standing normative, instrumental, and substantive rationales (Balog‐Way et al. 2020; Demeritt and Nobert 2014). If understanding Fischhoff's (1995) stages as a prescriptive guide, partnership may appear as a late‐stage refinement, something to consider once technical communication is optimized. But from more recent perspectives, defining and engaging partners is often the essential starting point for ethical and effective communication. Instead of risk communication *to* publics, more recent best practice methods promote risk communication *with* publics (Balog‐Way et al. 2020; Bier 2001; McComas et al. 2010).

**TABLE 2:** Modified Developmental Stages of Risk Management, developed from Fischhoff (1995).

All we have to do is get the numbers right
All we have to do is work as partners
All we have to do is communicate the numbers
All we have to do is explain what the numbers mean
All we have to do is show that similar risks were accepted in the past
All we have to do is show that it's a good deal
All we have to do is to be nice
All of the above

Relatedly, we also propose a rewording of Fischhoff's (1995) stages to be more inclusive, and complement later visualizations developed by Fischhoff (2015) to those seeking to use these as prescriptive models for risk communication design. While these changes may be minor, they better reflect the kind of partnership risk communicators should be striving for in their engagement with publics and allowing for multi‐way strategies beyond simple top‐down approaches and “us versus them” conceptualizations (see Table 2).

## 3 Fischhoff's (1995) Conclusions, 30 Years On

We next examine a selection of conclusions and recommendations from Fischhoff's (1995) commentary. Namely, we address Fischhoff's (1995) conclusions that risk communication: (1) Must be taken seriously; (2) must receive appropriate financial support; (3) needs more evidence of its effectiveness, and (4) should fulfil only part of the social contract between risk creators and those bearing the impacts of risks. In part, we reflect on progress made over 30 years on these conclusions and recommendations, observing how the current technological and societal zeitgeist may influence their progress and applicability.

One of the most fundamental conclusions Fischhoff (1995, 144) offers is that risk communication “has to be taken seriously”. This call remains urgent, and consistently repeated by risk communication researchers. In the context of polarization, misinformation, and declining public trust in science, undertaking multi‐way, participatory communication cannot be treated as an afterthought or a public relations exercise (Bouder 2025; Burger et al. 2022; Vollmer et al. 2025). Examples where risk communication research has been inappropriately or unsuccessfully incorporated into risk management strategies include Hurricane Katrina in the US (Cole and Fellows 2008), EU aspartame risk management (Lofstedt et al. 2011), and the COVID‐19 pandemic globally (Collins et al. 2020; Wardman 2020; Warren and Lofstedt 2021). Past failures to include appropriate risk communication activities have resulted in unforeseen negative consequences on risk outcomes, spread of misinformation and risk amplification, and erosion in public trust in expertise and risk managers (Comrie et al. 2019; Frewer 2003; Kasperson 2014; Lofstedt et al. 2011). Risk communication must be recognized more consistently as the core part of risk governance that Renn (2008) envisaged to mitigate future failures and promote trust in risk communicators and managers.

In the context of recognition and effective application, Fischhoff (1995) also underlines the importance of appropriate financial support for risk communication research and practice to achieve its goals. This is a consistent, if somewhat unsurprising, conclusion drawn throughout the last 30 years (Boholm 2019a; Kasperson 2014; Leiss 2004). Appropriately funding risk communication can allow for a wider reach, greater audience segmentation, and the ability to conduct truly inclusive participatory and dialogic methods that can be time‐consuming and expensive (Lundgren and McMakin 2018; Pidgeon 2021). Lofstedt (2003) argues that lack of funding for risk communication not only precipitates communication failures, but also restricts the ability to learn from past mistakes and anticipate future challenges through evaluating prior activities.

Certain technological developments, such as widespread internet use, social media and artificial intelligence (AI) have perhaps reduced certain costs of “doing” risk communication. This includes reaching a wider audience in inclusive ways due to greater overall use of fewer online platforms, appropriate message tailoring when using AI as a communications agent, or instantaneous messaging seen on social media platforms (Kessler et al. 2025; Mahl et al. 2025; Rains et al. 2014; Roth and Brönnimann 2013). Pre‐testing and post‐hoc evaluation, often called‐for in the risk communication literature (Lofstedt 2014; O'Connor et al. 2015), could simultaneously be made easier to do with the advent of AI, whether through the creation of “digital twins” that allow constant communication testing and practice with virtual representation of system, groups and/or individuals updated in real time (Kober et al. 2024).

Simultaneously, technological developments have made conducting risk communication more complex and potentially costly to “get right”, seen through continued audience fragmentation and the rise of echo chambers and filter bubbles in part as a result of social media and search engine algorithms (Kasperson et al. 2022; McIntyre 2018). Since the start of the COVID‐19 pandemic, online methodologies have become ubiquitous, allowing greater potential audience reach but also raising questions about their validity with the advent of greater automation, AI and its ability to provide valid and accurate information, and the rise of misinformation (Kessler et al. 2025; Mahl et al. 2025; Valenti et al. 2023). These emerging technologies also raise questions and concerns regarding their malign use in risk communication strategies of the future, and how one might address these (Krimsky 2007; Sellnow‐Richmond and Lukacovic 2024; van der Linden 2022).

To ensure appropriate benefits are accrued, the use of novel technologies also risks increasing financial and non‐financial costs arising from the need to provide greater training, plan more strategically, tackle new challenges such as misinformation, and continuously invest in new software and hardware, all while becoming more dependent on said technologies and associated bureaucracy for success (McComas 2004). Appropriate financial support therefore remains vital, particularly in the wake of changing and novel communications methods, technologies, and trends that require more pre‐testing and post‐hoc evaluation to ensure success.

Fischhoff (1995, 144) concludes that, when it comes to measuring and evaluating the effectiveness of risk communication, “the evidentiary record is less full than one would like”. While many conceptualizations and reviews of the evidence of “effective” or “impactful” risk communication have been offered over the decades (see Breakwell 2000; Rickard 2019; Zipkin et al. 2014), it can be challenging to measure or evaluate its impact *ex post*, particularly when at large scale or undertaken by central authorities (Boholm 2019b). This can be because the practice has been done proactively prior to crisis, or over a lengthy period, operational rationales such as the turnover of staff or lack of budgeting for program evaluation, or because it was used in conjunction with other risk management tools which can call into question causational relationships (Kasperson and Palmlund 1989; Rohrmann 1992). In addition, messengers who develop and evaluate risk communications campaigns could be hesitant in uncovering incompetence and failure in their own approaches, and the potential to face blame. High reliability organizations such as hospitals have begun to provide solutions to this issue by creating a culture of “collective mindfulness” that accepts negative evaluations that can help the organization continually improve (Chassin and Loeb 2013, 461; Weick and Sutcliffe 2011).

Some more recent attempts from authorities to develop and apply tools to evaluate the impact of risk communication activities have been carried out, such as the UK Government's Cabinet Office's (2011) *Communicating Risk Guidance* toolkit, the UK Food Standards Agency's (2020) *FSA Risk Communication Toolkit*, and the EFSA (2021) *Engagement Toolkit* for effective participatory processes. That said, evidence of these guides’ application in risk communication program are not as evident. Furthermore, evidence of publicly available data on the impact of risk communication strategies undertaken by relevant authorities on specific issues is scant, and in most cases would remain case‐ and/or nation‐specific and therefore difficult to apply universally. That said, more research is needed to explain why evaluation remains relatively rare despite decades of recommendations.

In recent years, the ability to use social media to communicate risk, and collect and monitor social media metrics, offers new data‐driven ways of understanding risk communication reach and public engagement (Overbey et al. 2017; Rains et al. 2014). While this evidence does not provide a causal link of the impact of risk communication on public knowledge, behavior change, policy support, and/or trust in authorities, it offers greater opportunity to collect data that can be used as evidence in evaluations of the impact of risk communication that was not possible before. This is seen through the collection of data‐driven metrics related to message success, such as number of clicks on, engagement with, and reach of a post, and evidence about the impact of communicating at different times of day that have assisted in testing and providing answers to many pre‐social media communications theories (Kim 2021; Overbey et al. 2017; Vos et al. 2018).

Researchers’ ability to freely access, collect, and analyze data and platforms’ Application Programming Interfaces (APIs) to evaluate the impact of risk communication strategies, while becoming more readily available, has seen roadblocks, as seen with the removal of these permissions on the Twitter/X platform in 2023 (Blakey 2024). This underlines that the “opening up” of information access and knowledge allowing us to better understand the impact of risk communication, depending on the use of novel technologies, is neither assured nor consistently increasing over time.

Overall, more evidence is needed to understand the impact of risk communication, particularly coming from central authorities and/or agencies. This evidence must include the impact of using different message attributes, messengers, modes of communication and platforms targeted at different audiences that have all changed drastically compared to the underlying assumptions of these in Fischhoff's (1995) article.

One of Fischhoff's (1995, 144) most important conclusions and recommendations is that “effective risk communication can fulfil part of the social contract between those who create risks […] and those who bear them”, but, that risk communication “should not […] paper over situations where people are getting a bad deal”. Despite this recommendation, a divide has persisted between risk communication practitioners and researchers as to what risk communication is for (Árvai 2014). Paternalistic attitudes about the purpose of risk communication and risk management among practitioners persist (Bouder 2025; Jardine and Driedger 2013; Perri 2000). Risk communication remains widely perceived by these groups, and those who fund or commission it such as risk managers and political actors, as a method of “correcting” public perceptions or “persuading” the public towards certain outcomes. This can be seen through simplistic solutions such as “educating the public” in isolation or “correcting misinformation” that show a lack of respect for those being “communicated to” (Árvai 2014; Ding 2025; Johnson 1999; Rickard 2019; Webler and Tuler 2021). Risk communication researchers’ emphasis on public engagement and dialogic processes as the recommended practice in the discipline has continued, yet not shaped external understandings of the discipline bar very few examples to the contrary (see Boholm 2019b). It is also important to reflect on potential contexts and circumstances in which persuasion and information provision are warranted and more appropriate for risk communicators to consider, such as when there is a clear ethical rationale for doing so (Thompson 2012), or where decision support is required for members of the public facing uncertainty in risk decision making, fulfilling risk communicators’ “duty to inform” (O'Connor et al. 2003; Spiegelhalter 2017, 34).

The continued (mis)perception of the purpose, methods, and value of risk communication by political and other actors underscores that there is still much to be done by the research community to emphasize the importance of respectful engagement with the public in strengthening the social contract in open democratic societies. Ways to address this could include supporting risk scientists’ understandings about how audiences process information, providing formal public communication training, promoting discussion about the “othering” of publics, and critically reflecting on the relationship between deficit model thinking and policy design, moving away from “simple problems” and “simple solutions” (Simis et al. 2016).

This imbalance between external perception of the discipline and reality makes us ask: What is risk communication for, and how is it perceived? Is it simply about managing the public response to decisions already made, persuading the public to undertake certain behaviors, and/or a final “step” of a risk management process in a truly instrumental sense (see Rickard 2019)? Or is it part of a broader democratic process that allows public voice and furnishes tools for more informed and confident decision‐making? Risk communication can also serve a conflict resolution goal; while this is not always realistic or legitimate, as Fischhoff (1995, 144) states, “the best‐case scenario for risk communication (and, indeed, risk management) is having fewer, but better conflicts”. Revisiting Fischhoff's (1995, 144) stages from these perspectives encourages us to question whether our current practices align with our ethical commitments, and reminds us that risk communication should not be used to “paper over situations where people are getting a bad deal”.

A further challenge to the ethical practice and evaluation of risk communication lies in the reality that research funders largely determine what funded research is undertaken, as well as the goals set, methods used, and teams’ makeup, based on funder priorities and (mis)perceptions of disciplines (Thelwall et al. 2023). Risk communication scholars are not immune to this influence, and may be perceived by potential knowledge users, such as central authorities, agencies, corporate entities, and research funding bodies, as fundamentally instrumental researchers to assist with persuasion or coercion goals. Care must be taken to ensure that central principles of risk communication, related to decision support and shared decision‐making, are not lost in the pursuit of research funding and the science‐policy interface.

## 4 Our Conclusions, 50 Years On

### 4.1 Repositioning of Importance of Partners

One of our central conclusions from Fischhoff's (1995) seminal article is the need to reposition the importance of partnership in risk communication due to the dramatic shift in how the discipline has developed in the last 30 years, centering the public at the heart of communication. Though we acknowledge that the intention of Fischhoff's developmental stages were a reflection on the field of risk communication and not set to be a prescriptive set of stages, the order of the stages and the numbering in the discussion may have inadvertently led to a prescriptive practice. Presented as one of the final stages in Fischhoff's (1995) paper, we believe that partnership must be centered in both theory and practice. Three decades of research, social and technological advancement (Balog‐Way et al. 2020; Bostrom et al. 2024; MacPherson‐Krutsky et al. 2025) show that identifying and working with partners is not just an advanced step, but should be a foundational starting point.

Meaningful risk communication cannot happen without shared ownership, inclusion, and responsiveness. It is not just about offering a seat at the table. It requires recognizing and valuing diverse public knowledge: Lived experience, cultural understanding, historical context, and activist critique. These perspectives challenge traditional notions of expertise and invite us to ask: Who gets to speak? Who is listened to? Who is left out? Technological advancements such as social media could enable more inclusive risk communication approaches today than in the time period of Fischhoff's (1995) original reflections, reaching wider and more specific audiences, with greater focus on tailoring to subgroups and including a wider range of voices into communication efforts (Kasperson et al. 2022; Rains et al. 2014). That said, these dynamics of power and partnership remain.

### 4.2 The Democratization of Risk Communication

Another conclusion arising from Fischhoff's (1995) stages concerns the language and democratization of risk communication (see Table 2). Since 1995, the information environment has changed significantly. Risk communication messages increasingly circulate through online spaces (Vos et al. 2018). The ability for risk communicators, managers, experts, and the public to communicate has become diffuse and arguably more equitable in the age of Web 2.0, with social media portrayed as lending anyone with a device a voice (Chew and Eysenbach 2010; Krimsky 2007). That said, engagement with social media is unevenly distributed in society, with powerful group interests continuing to dominate while the voiceless may yet remain voiceless (Feeney and Porumbescu 2021; Meyer and Wiley 2024). As a result, there is a broader diversity in information sources available to the public to receive and interact with. It can also be packaged by, and for, differing audiences that are more relevant, appropriate, and/or interesting to them (Ross and Rivers 2019). However, information shared in these formats on dispersed and often volatile digital platforms can be shaped by emotion, use differing discourse methods, produce amplificatory rapid feedback loops, and propagate misinformation (Comrie 2015; Gesser‐Edelsburg 2021; Krause et al. 2020). This shift demands a re‐evaluation of how risk communication approaches are conceived to account for democratized, informed publics with greater communicative agency.

Despite these challenges, the aims of risk communication remain consistent. Trustworthiness remains central, but is increasingly difficult to achieve in post‐trust and post‐truth eras (Lofstedt 2005; van der Linden and Löfstedt 2019). This prompts us to reflect: Are we really doing the things we say are important? Fischhoff (1995) is candid about the limitations of practice, noting that those who have progressed through the stages may still fail to apply them effectively. In our experience, many of the latter developmental stages remain largely aspirational. Though commonly cited in theory or policy, they are less frequently and unevenly embedded in practice (Kasperson et al. 2022).

This conflict, and increased ability and desire of publics to have a voice in risk communication brought about by the internet, social media, and evidence that it can improve decision‐making (Chew and Eysenbach 2010; Irvin and Stansbury 2004), also requires us to examine the language used within the field. Much of the language surrounding risk communication still carries undertones of scientific elitism. Terms such as “educating the public”, “behavior change”, or even “treating them nicely” suggest a dynamic in which the expert holds knowledge, and the public must be persuaded to accept it. Even the notion of “making them partners” implies a decision still held by those in power. If we are serious about collaboration, we must also be willing to interrogate how language reflects and reinforces unequal power relations. This speaks to our desire to reframe the developmental stages as a shared exercise (see Table 2). This discussion of scientific elitism and inter‐group respect between risk communicators and stakeholders requires greater exploration.

### 4.3 Participatory Approaches to Risk Communication

While it is easy to advocate the principles of partnership, transparency, and engagement in risk communication, meaningful enactment remains difficult. Many of the most progressive elements in Fischhoff's (1995) stages remain more visible in academic discussions than in applied settings. While often advocated for through models and frameworks, even in applied settings, participatory approaches to risk communication are often underfunded, under‐evaluated, or marginalized (Lofstedt 2003; Markon et al. 2013; Pfeiffer 2006; Webler and Tuler 2021). In practice, risk communication often reverts to data presentation, message testing, and public reassurance (Gochfeld 2022; Warren and Lofstedt 2021). These efforts, while well‐intentioned, rarely live up to the aspirations of shared decision‐making or true participatory engagement advocated by Webler and Tuler (2021) and Burdett (2024).

Participatory methodologies such as co‐production, deliberative processes, and mental models research offer promising ways to build genuine collaboration (Funtowicz and Ravetz 1993; Gatersleben et al. 2024; Lejano et al. 2021). But even these approaches can become performative if not supported by clear commitments to shared decision‐making and power redistribution (Gattinger 2023). This highlights the need to not just involve partners, but to clarify who they are, their role, and how influence is shared.

### 4.4 Subjectivity and Objectivity of Risk Communication

Finally, the subjectivity inherent in risk assessment, management, and communication, and their reception, is still inadequately addressed or acknowledged by experts in the field. Fischhoff (1995) did not address emotions, affective responses, heuristics and biases, and how these shape message reception. These factors are difficult to account for and can contribute to communication failures (Peters 2008; Sjöberg 2007; Xie et al. 2011). Tailored risk communication strategies may also (often purposely) evoke differing emotions and responses among different publics (Roeser 2012; Warren and Lofstedt 2021).

Risk is often presented as fully calculable and conveyable with precision and certainty; its meaning taken for granted in communication efforts (Aven 2025). As Fischhoff (1995) notes, risk analysis itself is a product of expert judgment, political context, and social values. Risk communication, then, is not simply the transfer of objective information, but a process shaped by uncertainty, contested knowledge, and competing priorities. It involves not only scientific accuracy, but interpretation, perspective, and trust. Acknowledging these limitations in both academic and applied settings is needed. While this may not align with public conceptualizations of and political desires for “certainty” and “facts” (Druckman 2015; Jasanoff 1986), accepting and communicating this reality strengthens risk communication's credibility through greater honesty, openness, and transparency.

## 5 Final Thoughts

Looking back, Fischhoff's (1995) seminal work on risk communication thirty years on has stood the test of time. It continues to be widely cited, used, and relied upon by risk communication scholars and practitioners. However, we suggest some updates to its framing 30 years on. We contribute to the development of the discipline by reframing risk communication, centered around partnership, democratization, and respect. Research developments, technology, and social change have transformed key elements of risk communication. New challenges, such as AI and digital platforms, present as yet unanswered, but crucial, questions for the future of the discipline. However, debates similar to those from 30 years ago persist, especially around funding risk communication, its value and purpose, and respect for the discipline.

Together, these reflections suggest that the future of risk communication depends not only on better techniques, but on greater critical and ethical awareness. It requires us to challenge the systems we work within, reflect on our own practices, train ourselves in social science, and build communicative approaches that are inclusive and reflexive to promote an optimistic future for risk communication research and practice and learn from history rather than repeating it. Fischhoff's (1995) article continues to resonate because it does not pretend the work is finished, even 30 years on in a more plugged‐in age. Instead, it reminds us that risk communication remains a process of learning, listening, and rethinking what our role should be.

## Conflicts of Interest

The authors declare no conflicts of interest.
